# Comparative analysis of aneurysm subtypes associated genes based on protein–protein interaction network

**DOI:** 10.1186/s12859-021-04513-w

**Published:** 2021-12-11

**Authors:** Ruya Sun, Yuan Zhou, Qinghua Cui

**Affiliations:** Department of Biomedical Informatics, School of Basic Medical Sciences, Key Laboratory of Molecular Cardiovascular Sciences of the Ministry of Education, Center for Non-Coding RNA Medicine, Peking University Health Science Center Beijing, Beijing, China

**Keywords:** Arterial aneurysm, Aneurysm subtype, Protein–protein interaction network, Disease driver gene, Programmed cell death

## Abstract

The arterial aneurysm refers to localized dilation of blood vessel wall and is common in general population. The majority of aneurysm cases remains asymptomatic until a sudden rupture which is usually fatal and of extremely high mortality (~ 50–60%). Therefore, early diagnosis, prevention and management of aneurysm are in urgent need. Unfortunately, current understanding of disease driver genes of various aneurysm subtypes is still limited, and without appropriate biomarkers and drug targets no specialized drug has been developed for aneurysm treatment. In this research, aneurysm subtypes were analyzed based on protein–protein interaction network to better understand aneurysm pathogenesis. By measuring network-based proximity of aneurysm subtypes, we identified a relevant closest relationship between aortic aneurysm and aortic dissection. An improved random walk method was performed to prioritize candidate driver genes of each aneurysm subtype. Thereafter, transcriptomes of 6 human aneurysm subtypes were collected and differential expression genes were identified to further filter potential driver genes. Functional enrichment of above driver genes indicated a general role of ubiquitination and programmed cell death in aneurysm pathogenesis. Especially, we further observed participation of BCL-2-mediated apoptosis pathway and caspase-1 related pyroptosis in the development of cerebral aneurysm and aneurysmal subarachnoid hemorrhage in corresponding transcriptomes.

**Supplementary Information:**

The online version contains supplementary material available at 10.1186/s12859-021-04513-w.

## Background

Arterial aneurysm describes a class of vascular diseases with focal dilation of arteries, that affects all arterial layers [1]. It might occur in various sites along the full length of arterial and present diverged morphologies, including saccular, fusiform, dissecting aneurysm and false aneurysm, sporadically or simultaneously. The classification of arterial aneurysm is mainly based on the anatomical location (e.g., abdominal aorta, thoracic aorta), the morphology and the complications (e.g., dissection, rupture). Accordingly, arterial aneurysm could be classified into different subtypes, such as abdominal aortic aneurysm (AAA), cerebral aneurysm (CA), thoracic aortic aneurysm (TAA), thoracic aortic aneurysm and dissection (TAAD), aortic aneurysm (AA), aortic dissection (AD), aneurysmal subarachnoid hemorrhage (ASH) [2]. Recent years, arterial aneurysm gradually becomes a common cardiovascular disease (CVD), especially among the elderly (≥ 65 years) [3, 4]. Today, the imaging-examination-based screening could dramatically improve early detection of aneurysm in population. However, typically aneurysm stays asymptomatic until a sudden rupture [5], which is usually fatal and with a mortality ranges up to 94% [3]. Even with repair surgery, the in-hospital mortality of ruptured aneurysm is still exceedingly high (e.g., 53.1% for AAA, 22% for TAA) [5]. At the same time, although progress has been achieved in aneurysm animal model development, understanding of the pathogenesis of specific mechanisms underlying various aneurysm subtypes is still poor. Thus, in this study, we utilize network-based approach to evaluate relationship between different aneurysm subtypes. Also, improved random walk method is applied to investigate potential driver genes of various subtypes. On this basis, we further study promising mechanisms contributing to aneurysm pathogenesis. Hopefully, these findings could facilitate understanding similarities and differences between different aneurysm subtypes, and accelerate the development of novel therapy for aneurysmal diseases.

## Results

### PPI Network-based similarity between different arterial aneurysm subtypes

Previously, we built the Aneurysm Gene Database (AGD) to collect article-supported aneurysm-gene associations [6]. We found that research progresses on various human aneurysm subtypes were not in sync, mainly focusing on certain popular subtypes like AAA and TAA [7]. While little is known about less prevalent subtypes like ASH and TAD, of which only a small number of disease-associated genes is known (<25 genes in AGD database). Although recent studies reveal that molecular, cellular pathology contributing to development of different aneurysm subtypes are quite distinct, they still share partial common characteristics [2, 8]. Therefore, we first compare the overall similarity of disease genes between various aneurysm subtypes in a biological network view.

**Fig. 1:**
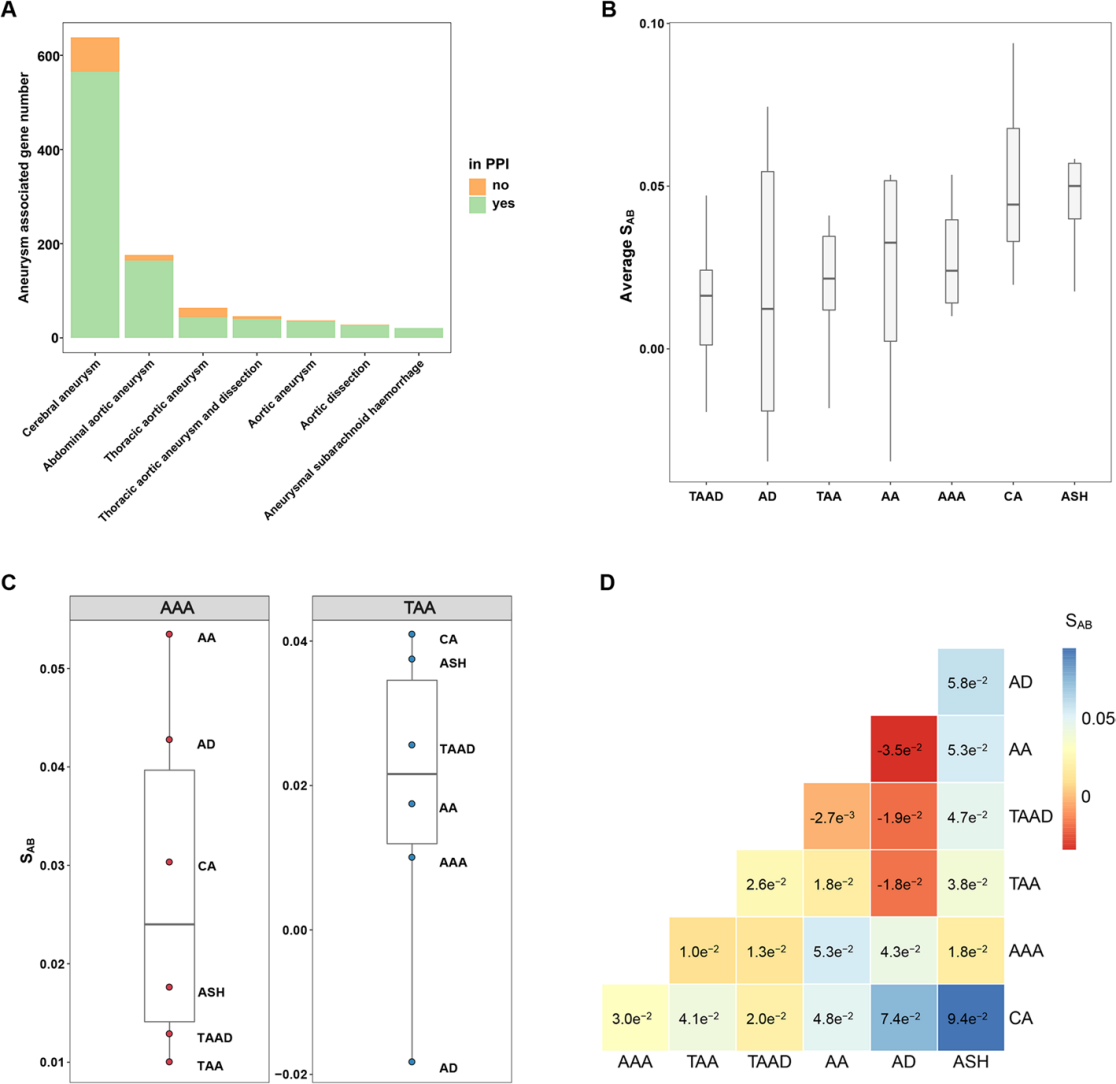
Overview of data source and network-based proximity between aneurysm subtypes. **a** Bar plot shows the number of genes associated with each aneurysm subtype recorded in the AGD database. The color of the bar corresponds to whether the genes could be found in the background PPI network or not. **b** Box plot presenting the SAB level between one aneurysm subtype and all other studied subtypes. The middle line of each box represents the corresponding median SAB value. **c** Scattered box plot describing the SAB value between AAA (left) or TAA (right) and all other studied subtypes. Each dot represents one aneurysm subtype. The names of corresponding aneurysm subtypes are labeled. **d** Heatmap describing the PPI network-based proximity (i.e. SAB values) between different aneurysm subtypes. The exact SAB values are also shown on each corresponding cell. AGD, the Aneurysm Gene Database; PPI, protein–protein interaction; AAA, abdominal aortic aneurysm; TAA, thoracic aortic aneurysm; TAAD, thoracic aortic aneurysm and dissection; AA, aortic aneurysm; AD, aortic dissection; ASH, aneurysmal subarachnoid hemorrhage; CA, cerebral aneurysm

Based on the notion that gene sets locating close in the protein-protein interaction (PPI) network more likely share common features, network-based proximity between aneurysm-subtype-associated gene sets was calculated. Human aneurysm-subtype-associated gene sets were firstly downloaded from the AGD, which was constructed to collect article proved aneurysm-associated genes by our group. Seven aneurysm subtypes, each of which has at least 20 known associated genes in AGD, were retained for subsequent analysis (seen in Fig. 1a). Network-based methodology was then applied to quantify relationship between different aneurysmal diseases.

Generally, the network-based proximity between various aneurysm subtypes was defined as S_AB_ (the separation score between aneurysm subtype A and aneurysm subtype B) and ranges from −3.5 × 10^-2^ (AD ~ AA) to 9.4 × 10^-2^ (CA ~ ASH). The average value of S_AB_ between one aneurysm subtype and other subtypes was also calculated. Among all the studied subtypes, ASH presents a highest average proximity to the other 6 subtypes (average S_AB_ = 5.1 × 10^-2^), suggesting high potential of ASH to own some distinct mechanisms. While the lowest average proximity to other subtypes was found in TAAD (average S_AB_ = 1.4 × 10^-2^; shown in Fig. 1b), indicating that TAAD is more likely to share some common mechanisms with the other subtypes. Besides, result reveals that the aneurysmal diseases with highest network-based similarity to popular aneurysmal diseases AAA and TAA are TAA (TAA ~ AAA, S_AB_ = 1.0 × 10^−2^) and AD (AD ~ TAA, S_AB_ = −1.8 × 10^-2^; shown in Fig. 1c) respectively. Moreover, a highest network-based proximity was finally found between AD ~ AA, AD ~ TAAD (S_AB_ = −1.9 × 10^−2^) and AD ~ TAA (shown in Fig. 1d). Above results suggest a high possibility to infer etiology and biology behind pathogenesis of AD from existing findings of AA, TAAD and TAA.

### Computationally predicted driver genes of various aneurysm subtypes

Current research on aneurysm pathogenic mechanisms and development of related biomarkers are constrained by limited number of early-stage patients and defective experimental animal model [9, 10]. To facilitate early disease diagnosis and treatment, it is important to explore potential biomarkers, especially promising driver genes, of aneurysmal diseases through computational method. We therefore applied network-based approach Driver_IRW to identify genetic drivers of each individual aneurysm subtype. Driver_IRW is a novel computational method, of which the basic assumption is that, in the interaction network, genes with higher degree have higher possibility to transit from upstream seed nodes [11]. Thus, genes with higher final random walk scores are more likely to be disease driver genes.

First, we constructed differential co-expression network [12] of each individual aneurysm subtype. To do this, we collected appropriate transcriptome profiles, which include samples from both aneurysm patients and healthy control, of previously filtered 7 aneurysm subtypes from the Gene Expression Omnibus (GEO) database (https://www.ncbi.nlm.nih.gov/geo/) [13]. Expression profiles of all subtypes except TAAD were acquired, thus 6 subtypes except TAAD were kept for subsequent analysis (Additional file 1: Table S1). Using expression data of patients and healthy donors, differential co-expression network (DCN) of each aneurysm subtype was constructed. By taking intersection edges of background PPI network [14], we further simplified relative DCN for different aneurysm subtypes (Additional file 1: Table S2). Next, aneurysm subtype-associated gene sets were extracted from the AGD database as the seed nodes. Using respective DCNs, seed nodes and expression matrices acquired before as input of Driver_IRW, final random walk scores of all nodes (genes) within each aneurysm subtype were calculated. The top 100 ranked genes with highest final scores were preliminarily listed as potential candidate driver genes.

**Fig. 2:**
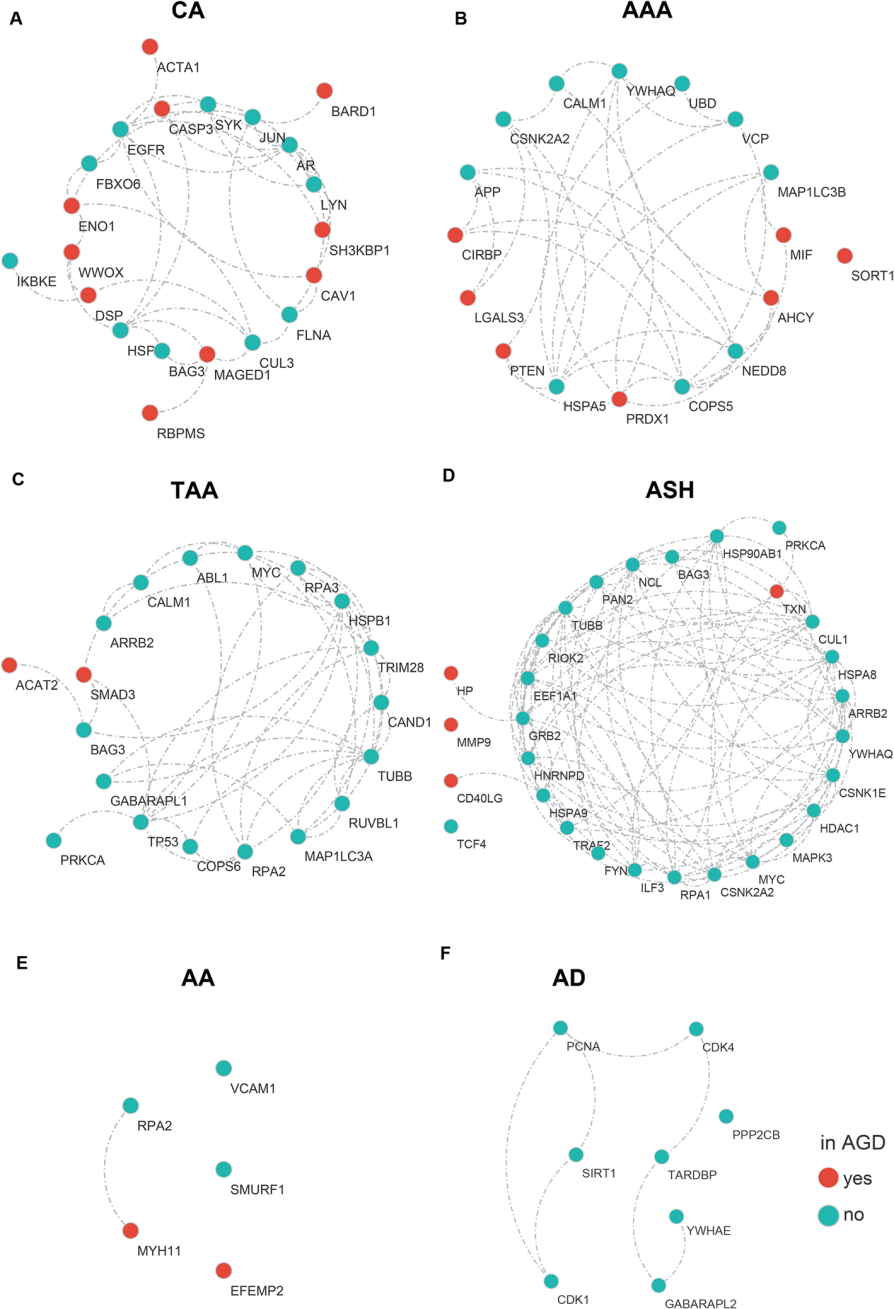
Predicted driver genes of each individual aneurysm subtype. The network diagrams depict the most promising candidate driver genes of **a** CA, **b** AAA, **c** TAA, **d** ASH, **e** AA and **f** AD. Color of the nodes indicates whether corresponding gene-aneurysm association could be found in the AGD database or not

To further narrow down the candidates and improve the prediction reliability, differential expression genes (DEGs) were calculated for every aneurysm subtype. Based on the transcriptome profiles of the 6 aneurysm subtypes, genes with significantly differential expression in aneurysm samples versus matched control were identified for each aneurysm subtype separately, with an adjusted *p*-value < 0.05. Intersection of top 100 ranked genes and DEGs was eventually filtered out as the final list of candidate driver genes for each subtype (seen in Fig. 2, Additional file 1: Table S3).

One straightforward approach to check the validity of the predicted driver genes is to compare the predictions with disease genes reported in literature. It is obviously that part of the driver genes had already been collected in the AGD database. However, considering that the AGD database was constructed in 2018, a number of new researches on aneurysmal diseases have come out in the past few years. Thus, for each candidate driver gene, we manually checked if there is any newly-published article supporting the associations between the predicted driver genes with corresponding aneurysm subtype. Results confirm that some of these genes have indeed been recently proved to be associated with aneurysm in human sample or animal model (Additional file 1: Table S4) even though they were not recorded in previous AGD database. Therefore, the recent literature evidence suggests the usefulness of the predicted driver gene list for the further investigation of aneurysmal diseases.

### Functional enrichment analysis of driver genes suggest the involvement of ubiquitination in aneurysm pathology

**Fig. 3:**
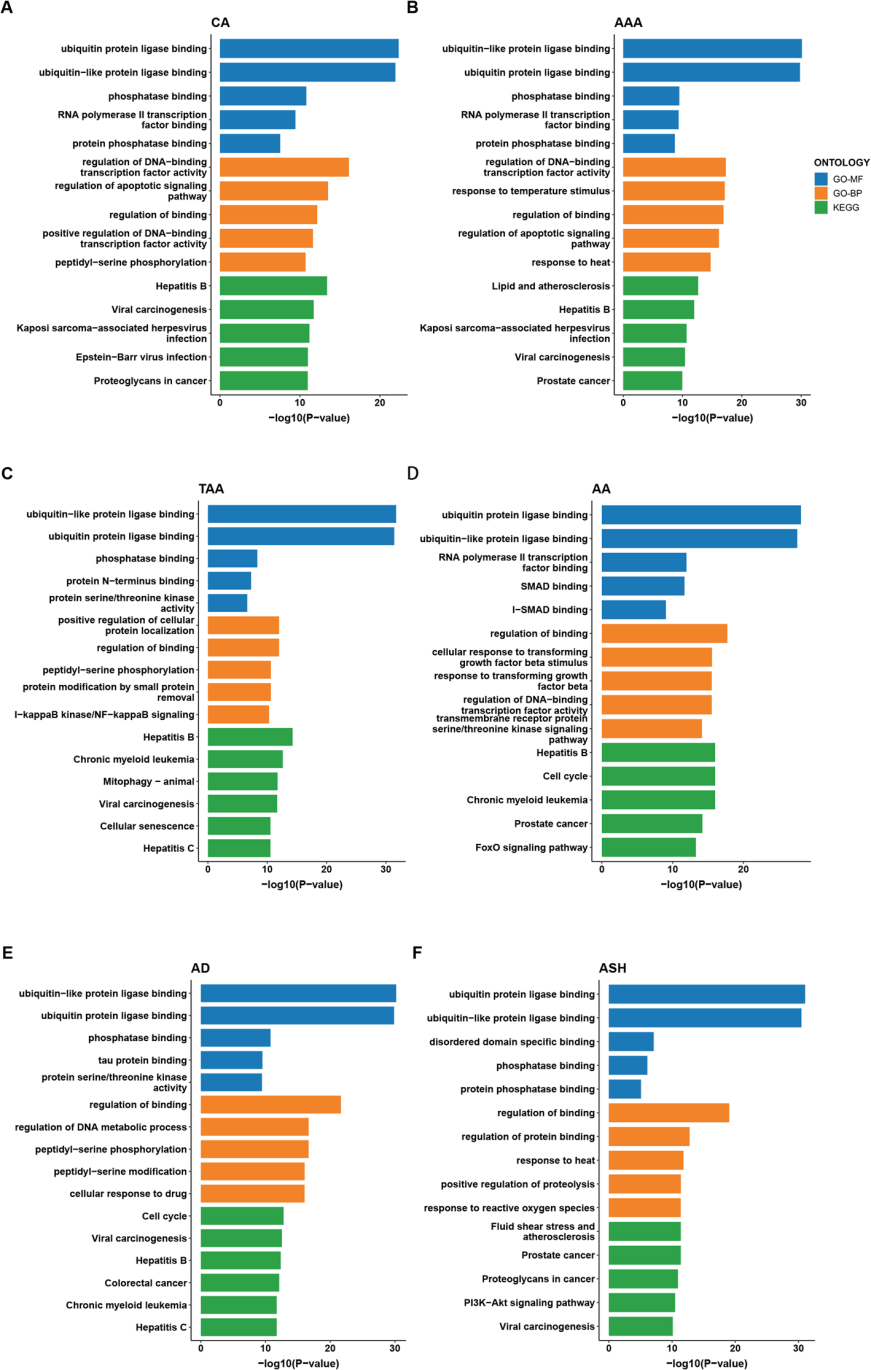
Functional enrichment analysis of potential drivers for each aneurysm subtype. The bar plots display functional enrichment result of potential drivers with top 100 ranked final random walk score for **a** CA, **b** AAA, **c** TAA, **d** AA, **e** AD and **f** ASH. The bar color represents different enrichment items, including Gene Ontology molecular function (GO-MF), Gene Ontology biological process (GO-BP), and Kyoto Encyclopedia of Genes and Genomes (KEGG)

Next, we tried to dig up novel mechanisms underlying aneurysm pathogenesis. Functional enrichment of above acquired top 100 ranked candidate drivers was performed for each aneurysm subtype respectively. Using the ‘clusterProfiler’ R package, the most enriched Gene Ontology (GO) and Kyoto Encyclopedia of Genes and Genomes (KEGG) functional category terms were obtained for different subtypes (Fig. 3). Obviously, enrichment result suggests an extensive involvement of ubiquitination associated pathways, including ubiquitin protein and ubiquitin-like protein binding activity, in all 6 studied subtypes. Through literature searches we found that the participant of ubiquitination related proteins (e.g. UCHL1, RNF213, UBR3, ARIH1) in pathogenesis of above-mentioned human aneurysm subtypes (ASH, AAA, CA, AA, AD) has been widely reported before [15–21]. As we already know, the ubiquitin-proteasome system (UPS) is a critical pathway in eukaryotic cells which functions through degrading cytosolic and nuclear proteins. By regulating relevant cell proteins, the UPS is essential for various important biological processes like inflammation and phenotypic changes [22]. Thus, we presumed that ubiquitin binding activity might play a role aneurysm development through regulation of vascular inflammation and vascular smooth muscle cells (VSMCs) phenotype switch. Also, some articles report that variants of ubiquitination associated genes might predispose to aneurysmal diseases, for example RNF213 variants for CA/ASH [20]. Therefore, we speculated that ubiquitination might be a mutual risk factor for various aneurysmal diseases.

### Involvement of apoptotic programmed cell death in aneurysm development

**Fig. 4:**
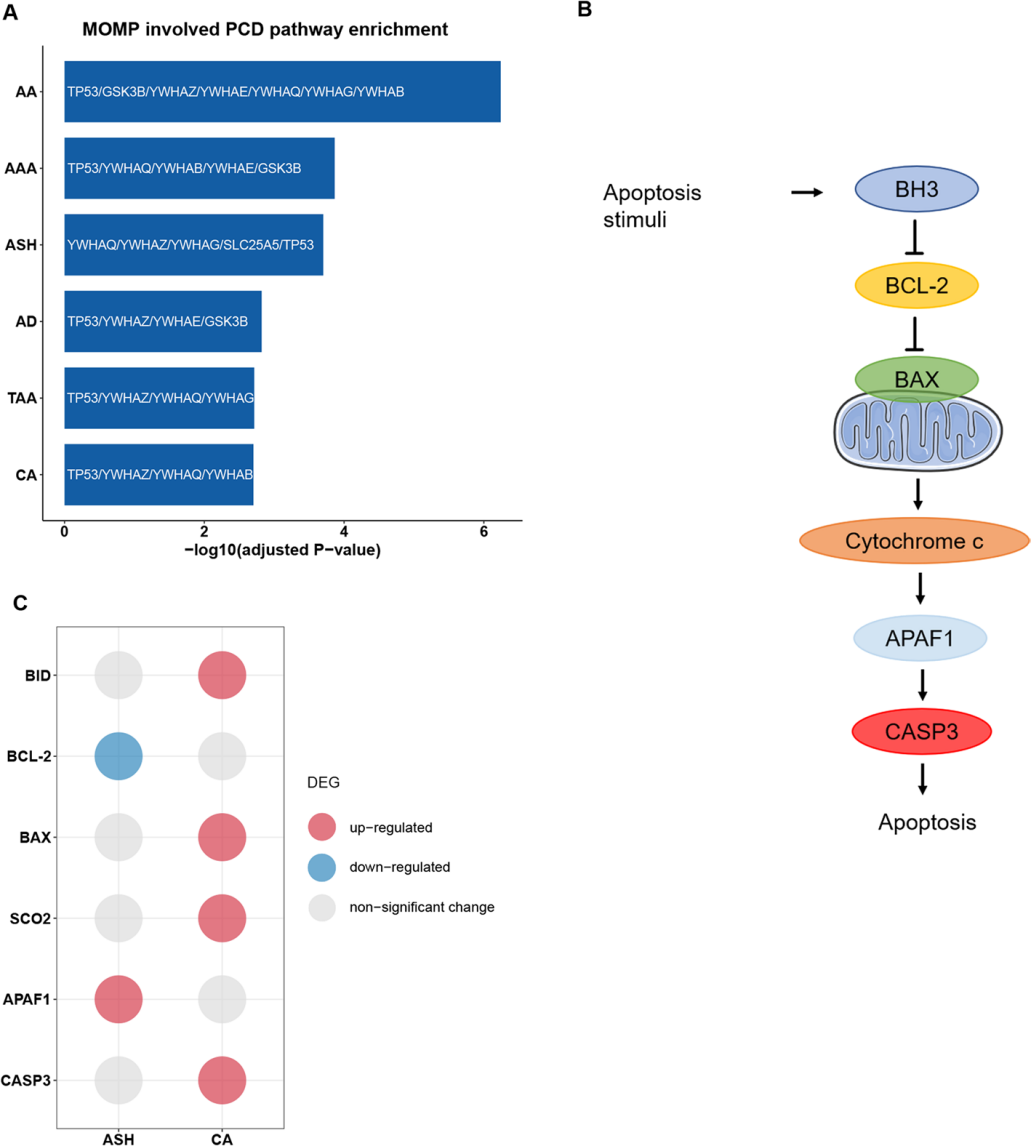
Extensive involvement of the ‘intrinsic’ apoptosis pathway in aneurysm pathogenesis. **a** The bar plot shows the comprehensive enrichment of the mitochondrial outer membrane permeabilization (MOMP) involved programmed cell death (PCD) pathway in aneurysmal diseases. Genes associated with this enriched pathway are labeled on corresponding bars. **b** Schematic plot illustrating the classic ‘intrinsic’ apoptosis pathway. **c** Bubble plot depicting the ‘intrinsic’ apoptosis pathway associated genes with significantly different expression level between human ASH or CA and healthy control. The color of each bubble indicates different change modes of the expression level. DEG, differential expression genes

Also, functional enrichment result reveals a consistent association with mitochondrial outer membrane permeabilization (MOMP) involved programmed cell death (PCD) pathway of all investigated aneurysm subtypes (seen in Fig. 4a). As evolutionally conserved suicide process of cell, PCD is critical to survival, development and disease pathogenesis in human. Up to now, several modes of PCD have been reported, including apoptosis, necroptosis, autophagy and pyroptosis [23]. It is well acknowledged that MOMP plays a critical role in apoptosis. In human, the apoptosis signaling is mainly classified into the ‘extrinsic’ pathway and the ‘intrinsic’ (also called the mitochondrial) pathway [24]. When a cell receives apoptosis stimulus, MOMP would commit it to die by the ‘intrinsic’ way. The process is predominately driven by B-cell lymphoma protein-2 (BCL-2) family members, including anti-apoptotic proteins (e.g. BCL-2), pro-apoptotic effectors (e.g. BAK, BAX) and BH3-only proteins (e.g. BID). Regulated by the BCL-2, MOMP would be induced in the mitochondrial outer membrane, promoting subsequent release of cytochrome c from mitochondrial to the cytosol. Once translocated to the cytosol, cytochrome c would interact directly with apoptotic protease-activating factor-1 (APAF1), forming the apoptosome complex. The apoptosome would then activate the initiator caspase-9, leading to downstream activation of executioner caspases (e.g. caspase-3, caspase-7). In response, executioner caspases would cleave and activate other executioner caspases, inducing apoptosis cascade and amplifying the apoptotic signaling (seen in Fig. 4b) [23, 25, 26].

Previously published researches have observed elevation of mitochondrial-apoptosis-dependent cell death of VSMCs in human aneurysm tissue (TAAD, AAA), provoking VSMC loss and aneurysm progression [27, 28]. In this research, result of afore performed DEG analysis highlights a potential involvement of BCL-2-mediated mitochondrial apoptosis pathway which functions through downstream activation of caspase-3 in pathogenesis of ASH as well as CA. In human ASH samples, significant decreasing expression of BCL-2 (adjusted *p*-value = 7.27 × 10^-5^) and increasing expression of APAF1 (adjusted *p*-value = 3.01 × 10^-2^) were identified compared to that in healthy samples. Also, higher expressions of genes including BID (adjusted *p*-value = 1.72 × 10^-5^), BAX (adjusted *p*-value = 3.89 × 10^-8^), SCO2 (adjusted *p*-value = 2.68 × 10^-5^) and CASP3 (adjusted *p*-value = 3.43 × 10^-2^) were observed in tissue sampled from CA patients than that from healthy donors (shown in Fig. 4c). Among them, SCO2 is a cytochrome c oxidase assembly factor which could facilitate reactive oxygen species (ROS) generation and positively regulate apoptosis signaling [29]. Based on these findings, we speculate that the BCL-2-mediated ‘intrinsic’ apoptosis pathway might widely function in various aneurysmal diseases, promoting VSMCs phenotype switch, thus might be a novel therapeutic target for aneurysm prevention and treatment.

### Pyroptotic programmed cell death and aneurysm pathogenesis

**Fig. 5:**
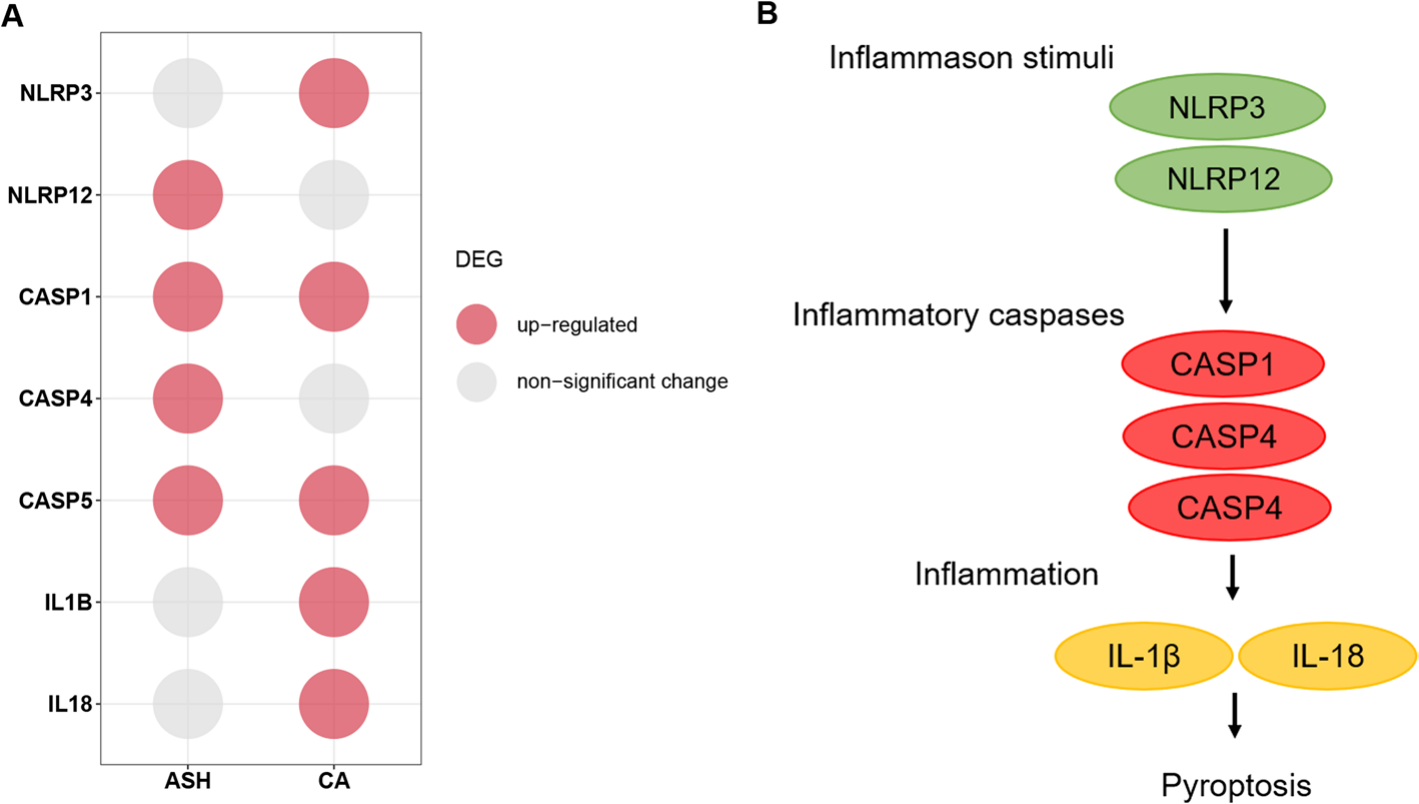
Participant of pyroptotic cell death pathway in development of ASH and CA. **a** Bubble plot displaying the pyroptosis pathway associated genes that are significantly differentially expressed between human ASH or CA and healthy control. The bubble color represents different change modes of the expression level. **b** Schematic diagram illustrating the pyroptosis signaling pathway

Further, we investigated whether other forms of PCD contribute to aneurysm pathogenesis, considering they share some common features and signaling molecules. DEG result reveals a significant expression level change of key pyroptosis-regulation genes in human ASH and CA samples (seen in Fig. 5a). As we already know, similar to apoptosis, pyroptosis is an inflammatory PCD pathway, which was proposed by Cookson and Brennan in 2001. While different from typical apoptosis, pyroptosis is characterized by BCL-2-resistant, caspase-3-independent and caspase-1-dependent [30]. Canonically, the pyroptosis process is activated with formation of inflammasome sensors in response to danger signal stimulus. The inflammasomes are intracellular multiprotein complexes, which mainly nucleate around Nucleotide-binding, Leucine-rich Repeat containing proteins (NLRP) family member, such as NLRP3, NLRP12 [31]. Inflammatory caspase (human caspase-1/4/5) would then be recruited and activated, leading to pyroptosis. Activation of caspase-1 would then lead to maturation and secretion of pro-inflammatory factors (IL1β, IL18) and to pyroptosis (seen in Fig. 5b).

Recently, contributions of pyroptotic cell death to development of human AAA and AD have been reported [32–34]. Here we observed significant upregulation of NLRP3 (adjusted *p*-value = 6.64 × 10^−4^), CASP1 (adjusted *p*-value = 1.25 × 10^−3^), CASP5 (adjusted *p*-value = 1.54 × 10^−2^), IL1B (adjusted *p*-value = 9.44 × 10^−5^) and IL18 (adjusted *p*-value = 7.01 × 10^−5^) in human CA samples compared to that in healthy control. Similarly, significant increased expression level of genes including NLRP12 (adjusted *p*-value = 4.77 × 10^−3^), CASP1 (adjusted P-value = 2.75 × 10^-2^), CASP4 (adjusted *p*-value = 8.90 × 10^−3^) as well as CASP5 (adjusted *p*-value = 1.85 × 10^-2^) was identified in human ASH samples (Fig. 5a). Altogether, these findings suggest a general participation of canonical NLRP-3-dependent pyroptosis as well as non-canonical pyroptosis in multiple aneurysmal diseases, indicating great potential of pyroptosis as novel target for aneurysm prevention.

## Discussions

Arterial aneurysm is a common class of cardiovascular disease with high prevalence, especially in the elderly. In men aged between 75 and 85, the prevalence could be up to 12.5% [5]. To date, diagnosis of aneurysm is mainly dependent on radiological and ultrasonography examination. Although community-based screening of aneurysm is highly recommended and has been proved to be able to greatly decrease relevant mortality, the estimation screening rate is still extremely low (~1.4%) due to lack of awareness [35]. In most cases, asymptomatic aneurysm is diagnosed incidentally during imaging examination for other unrelated diseases [9]. Most aneurysm cases remain undiagnosed until a sudden burst of rupture, which is highly lethal, relevant mortality could go over 90%. Thus, to prevent aneurysmal death from mortal complications, early diagnosis and dynamic monitoring is rather necessary, especially for early-stage asymptomatic aneurysm.

In this research, we tried to explore potential disease driver genes for different aneurysmal diseases using computational method. First, we studied relationship between various aneurysm subtypes, based on the assumption that similar subtypes might share partial mechanisms. The similarity between different subtypes was quantified using proximity in PPI network. Result shows a highest average similarity to other subtypes in TAAD and the lowest average similarity in ASH, indicating that ASH is more like to have some unique mechanisms while TAAD might share more common mechanisms. Also, we found a highest similarity between AA ~ AD, TAAD ~ AD and TAA ~ AD. Thus, we presume that AD might share a few common characteristics with AA, TAAD and TAA. So, it is highly recommended to infer potential pathology of AD by referring known mechanisms of TAAD, TAA and AA in future research.

Besides, using improved random-walk-based methodology Driver_IRW, we predicted the most potential promising driver genes for each aneurysm subtype. Genes with the final random score ranked top 100 were selected as the initial candidates for each subtype. To further improve the reliability of the prediction result, list of genes differentially expressing between human aneurysm tissue and healthy control was calculated for each subtype. Combined the top 100 ranked candidate driver genes and the significant DEGs (adjusted *p*-value < 0.05) and took the intersection, we obtained the final list as the most promising driver genes for each studied aneurysmal disease. Part of the gene-aneurysm associations in the final list has been collected in the AGD database, and another part of them have been reported in recent years, together support the reliability of the prediction result. These candidate drivers might be potential biomarkers and optional therapeutic target for diagnosis as well as treatment of aneurysmal diseases.

Furthermore, to dig out novel mechanisms, GO and KEGG functional enrichment of afore-obtained potential candidate drivers with final random walk score ranked top 100 was performed for each aneurysm subtype. Result suggests a general role of ubiquitination associated pathways, including ubiquitin protein binding activity and ubiquitin-like protein binding activity, in all 6 studied subtypes. Through literature survey, we found that involvement of ubiquitination relevant pathways in aneurysm pathogenesis has been reported before in ASH, AAA, CA, AA and AD [15–21]. Thus, it is highly possible that ubiquitination extensively participates in progress of different aneurysmal diseases, through regulation of vascular inflammation and VSMCs phenotypic changes.

Moreover, enrichment analysis reveals a potential role of MOMP involved programmed cell death in development of ASH and CA. Confirmed by the DEG analysis result, BCL-2-mediated mitochondrial apoptosis pathway associated molecules positively promote apoptotic cell death in ASH and CA samples. It is well acknowledged that, in aneurysm pathogenesis, VSMCs loss as well as extracellular matrix degradation work together to contribute to the degeneration of medial arterial layer, destruction of arterial wall morphology and subsequent aneurysm formation [2, 36]. Therefore, we speculate that the ‘intrinsic’ apoptosis might promote aneurysm development through activating VSMCs death. Further, we found another mode of programmed cell death, pyroptosis, might also participant in progress of ASH and CA. Extracting DEG result of pyroptosis associated genes, we found an overall expression level upregulation of related genes in human aneurysm tissues compared to matched control. Thus, pyroptotic cell death might also involve in aneurysm pathology. Pyroptosis might function in a way similar to apoptosis, by positive regulation of VSMCs death. Besides, a number of researches indicate an important role of chronic vascular inflammation in aneurysm pathogenesis. It is well recognized that chronic vascular inflammation facilitates the destruction of aortic media and the dysfunction of VSMCs [10]. Therefore, downstream activation of critical pro-inflammation factors like IL1B and IL18 in pyroptosis process [30], might also play a role in aneurysm progression. In addition, literature search verified that involvement of both mitochondrial apoptosis and pyroptosis have been reported before in other aneurysm subtypes. Altogether, above findings indicate a promising value of mitochondrial apoptosis as well as pyroptosis, the two classic programmed cell death modes, as therapeutic targets for treatment of ASH and CA.

## Conclusions

In this study, we evaluate relationship between different aneurysm subtypes and find a highest PPI-network-based similarity between AD and AA, TAA, TAAD, suggesting a potential to infer novel mechanisms contributing to AD on the reference of existing understanding of AA, TAA and TAAD. Utilizing random-walk-based approach and DEG analysis, we obtain list of the most promising drivers for each of the 6 studied aneurysmal diseases. We hope this part of results could facilitate development of biomarkers and therapeutic targets for aneurysm, and help reduce the time and money cost of future research. We also reveal an extensive involvement of ubiquitination pathway in various aneurysm subtypes. Ubiquitination process might promote aneurysm formation through regulation of vascular inflammation and VSMCs phenotype change. Further, intrinsic apoptosis and pyroptosis, the two classic pathways of programmed cell death, may also participate in pathogenesis of various aneurysm subtypes. These two PCD pathways may function through promoting VSMCs death and activating inflammation, leading to VSMC loss and subsequent aneurysm development.

Still, we have to point out a few limitations of our research. First, conclusions of this research are made totally based on computational calculation. Both in vivo and in vitro experiments are still needed to verify the hypotheses we have proposed. Second, only 7 aneurysm subtypes have been studied here, and a number of aneurysmal diseases are not included because of the limited understanding at present. Future research focusing on the less popular subtypes will definitely help improve our predictions and also the understanding of the molecular mechanisms of aneurysmal diseases.

## Methods

### Datasets for aneurysm-associated genes and transcriptome profiles

Gene set associated with different human aneurysm subtypes were obtained from the AGD database [6]. 1179 aneurysm-gene associations covering 27 aneurysmal diseases have been recorded in AGD. To ensure research quality, aneurysm subtypes with less than 20 associated genes recorded in AGD were excluded in this research. By procedure, only 7 human aneurysm subtypes with sufficient data (CA, AAA, TAA, TAAD, AA, AD, ASH) were included in the subsequent analyses.

Transcriptome profiles of human aneurysm subtypes were accessed from NCBI’s GEO database. First, for all above-mentioned aneurysm subtypes, the primary candidate GEO datasets were screened using search item “(aneurysm subtype) AND ‘Homo sapiens’[porgn:_txid9606]”. Considering the need to construct differentially co-expressed network, only GEO series including samples from both aneurysm patients and matched healthy donors were retained. For each aneurysm subtype, the GEO dataset of the largest sample size was finally chosen for following analysis. As a result, except TAAD (of which the transcriptome profile could not be found in GEO database), we obtained transcriptome profiles of all other 6 aneurysm subtypes from GEO database.

### Network-based proximity between aneurysm subtypes

We first downloaded the background PPI network, which was built on reference of 15 popular databases, from previously research [14]. This network including 217160 interactions (edges) and 15970 genes (nodes). The gene sets of different aneurysm subtypes were further filtered by retaining only genes which could be found in this background network.

Network-based proximity was measured using the separation method [37] to quantify similarity between different human aneurysm subtypes [14]. Taking above-mentioned background network as reference, we first extract gene set of each aneurysm subtype separately. Then, the separation score between aneurysm subtype A and aneurysm subtype B was calculated as follows:

$${\text{s}}_{{{\text{AB}}}} \equiv \left\langle {{\text{d}}_{{{\text{AB}}}} } \right\rangle - \frac{{\left\langle {{\text{d}}_{{{\text{AA}}}} } \right\rangle + \left\langle {{\text{d}}_{{{\text{BB}}}} } \right\rangle }}{2}$$

where $\left\langle {{\text{d}}_{{{\text{AB}}}} } \right\rangle$ corresponds to the mean shortest path length between A–B node pairs, while $\left\langle {{\text{d}}_{{{\text{AA}}}} } \right\rangle$ and $\left\langle {{\text{d}}_{{{\text{BB}}}} } \right\rangle$ represent the mean of shortest distance between all nodes within A gene set and B gene set separately. For genes associated with both aneurysm subtype A and B, their distribution to $\left\langle {{\text{d}}_{{{\text{AB}}}} } \right\rangle$ should be zero. Therefore, the smaller ${\text{s}}_{{{\text{AB}}}}$ suggest the harder to separate gene set A and B in the network and higher similarity between aneurysm subtypes A and B. Here, the shortest path in PPI network was calculated based on unweighted breadth-first search algorithm, using ‘get.shortest.paths’ function of igraph R package (v1.2.6) [38].

### Prediction of potential aneurysm associated genes

To predict the most promising driver genes of various aneurysm subtypes, a state-of-the-art random-walk-based approach Driver_IRW was employed by using the R package ‘Pijing09/Driver_IRW’ [11]. Driver_IRW was developed to identify and prioritize driver genes which contribute to disease pathology based on transcriptome profile and interaction network. The basic assumption is that genes with higher final random walk scores in the interaction network tend to have functions similar to the seed genes, and thus are more likely to be the driver genes. We first constructed DCNs for different aneurysm subtypes separately. Taking aforementioned transcriptome profiles as the reference, Pearson correlation coefficients and corresponding *p*-values of all gene pairs in aneurysm samples and matched control samples were calculated separately. Afterwards, gene pairs with *p*-value < 0.05 in either aneurysm samples or matched control samples were selected as differential co-expression edges (DCEs). Intersection between interacting gene pairs in background PPI network and filtered DCEs was finally used to build the DCNs, with weight of all edges assigned as 1. Next, intersection of known aneurysm subtype-associated genes recorded in AGD and genes in the corresponding DCN were extracted as the seed genes for each aneurysm subtype. Using DCNs, seed genes, gene expression matrix of aneurysm subtype as the input of Driver_IRW (default damping factor 0.85 was used), random walk score of each gene in different aneurysm subtypes were calculated to measure function similarity of each gene to known seed genes. The final random walk score was applied to evaluate potential of each gene as the driver gene for the corresponding aneurysm subtype.

### Differential expression gene analysis and functional enrichment analysis

To further filter potential driver genes of different aneurysm subtypes, DEG analysis was performed. Using limma R package (v3.40.6) [39], genes expressing in significantly different level between aneurysm group and control group were identified (empirical Bayes method, Benjamini-Hochberg adjustment, adjusted *p*-value < 0.05).

GO functional enrichment analysis was made subsequently to explore roles played by candidate biomarkers. Using ‘enrichGO’ function of clusterProfiler R package (v3.12.0) [40], with ‘org.Hs.eg.db’ subject as background annotation, corresponding enriched GO terms were accessed. Results of molecular function (MF) and biological process (BP) subontologies were retained. Using ‘enrichKEGG’ function of clusterProfiler R package, with ‘hsa’ as background organism, we obtained enriched terms of KEGG.

## Supplementary Information

**Additional file 1:** Supplementary tables.

## Data Availability

All the expression profiles were extracted from the public database GEO (https://www.ncbi.nlm.nih.gov/geo/). All the articles supporting the findings of this study can be found in the public resource PUBMED (https://pubmed.ncbi.nlm.nih.gov/). The accession numbers and the article IDs can be found in the Additional file 1.
