# Supplementary material for: Comparative analysis of aneurysm subtypes associated genes based on protein–protein interaction network
Source: BMC Bioinformatics. 2021 Dec 11;22:587. doi: 10.1186/s12859-021-04513-w (PMC8665538; doi:10.1186/s12859-021-04513-w)
Supplement: Supplementary file 1 — Additional file 1: Supplementary tables. [file 12859_2021_4513_MOESM1_ESM.docx]

**Supplementary materials**

**Comparative Analysis of Aneurysm Subtypes Associated Genes Based on Protein-Protein Interaction Network**

Ruya Sun^1 *^, Yuan Zhou^1^, Qinghua Cui^1 *^

^1^Department of Biomedical Informatics, School of Basic Medical Sciences, Key Laboratory of Molecular Cardiovascular Sciences of the Ministry of Education, Center for Non-coding RNA Medicine, Peking University Health Science Center Beijing, China

Correspondence to:

Ruya Sun

sunruya@pku.edu.cn

Qinghua Cui, PhD, Professor

cuiqinghua@hsc.pku.edu.cn

**Table S1. Data source of collected aneurysm subtype transcriptome profile**

| Aneurysm subtype | GEO data source |
| --- | --- |
| Cerebral aneurysm (CA) | GSE75436 |
| Abdominal aortic aneurysm (AAA) | GSE57691 |
| Thoracic aortic aneurysm (TAA) | GSE9106 |
| Aortic aneurysm (AA) | GSE140947 |
| Aortic dissection (AD) | GSE52093 |
| Aneurysmal subarachnoid haemorrhage (ASH) | GSE36791 |

**Table S2. Simplified differential co-expression network scale of different aneurysm subtypes**

| Aneurysm subtype | Number of nodes | Number of edges |
| --- | --- | --- |
| CA | 10800 | 89786 |
| AAA | 13615 | 113470 |
| TAA | 11752 | 146498 |
| AA | 11407 | 147652 |
| AD | 10074 | 67164 |
| ASH | 11480 | 108480 |

**Table S3. Candidate drivers of different aneurysm subtypes**

| Aneurysm subtype | Candidate driver | Final random score | include in AGD | |
| --- | --- | --- | --- | --- |
| CA | CUL3 | 0.299949208 | not include |  |
| CA | JUN | 0.104067822 | not include |  |
| CA | CAV1 | 0.072144592 | include |  |
| CA | WWOX | 0.071405449 | include |  |
| CA | EGFR | 0.068103196 | not include |  |
| CA | FBXO6 | 0.065760017 | not include |  |
| CA | BAG3 | 0.057906555 | not include |  |
| CA | CASP3 | 0.03470832 | include |  |
| CA | IKBKE | 0.03422189 | not include |  |
| CA | ACTA1 | 0.03408536 | include |  |
| CA | DSP | 0.033472907 | include |  |
| CA | RBPMS | 0.031291461 | include |  |
| CA | MAGED1 | 0.030358253 | include |  |
| CA | SH3KBP1 | 0.029056596 | include |  |
| CA | LYN | 0.028334496 | not include |  |
| CA | HSPB1 | 0.027754486 | not include |  |
| CA | ENO1 | 0.026001613 | include |  |
| CA | AR | 0.025090506 | not include |  |
| CA | BARD1 | 0.023171825 | include |  |
| CA | FLNA | 0.022960145 | not include |  |
| CA | SYK | 0.022933113 | not include |  |
| AAA | APP | 0.144597715 | not include |  |
| AAA | PTEN | 0.037184994 | include |  |
| AAA | COPS5 | 0.036829769 | not include |  |
| AAA | PRDX1 | 0.036631149 | include |  |
| AAA | CIRBP | 0.031288798 | include |  |
| AAA | AHCY | 0.027474721 | include |  |
| AAA | VCP | 0.020378878 | not include |  |
| AAA | MIF | 0.018737606 | include |  |
| AAA | YWHAQ | 0.018427177 | not include |  |
| AAA | SORT1 | 0.016264965 | include |  |
| AAA | NEDD8 | 0.013225132 | not include |  |
| AAA | CALM1 | 0.012924598 | not include |  |
| AAA | LGALS3 | 0.01203547 | include |  |
| AAA | UBD | 0.011567357 | not include |  |
| AAA | HSPA5 | 0.011044435 | not include |  |
| AAA | CSNK2A2 | 0.010013877 | not include |  |
| AAA | MAP1LC3B | 0.009371367 | not include |  |
| TAA | SMAD3 | 0.103189213 | include |  |
| TAA | ACAT2 | 0.026775167 | include |  |
| TAA | TP53 | 0.026762488 | not include |  |
| TAA | MYC | 0.013561552 | not include |  |
| TAA | CAND1 | 0.012294252 | not include |  |
| TAA | MAP1LC3A | 0.008082646 | not include |  |
| TAA | GABARAPL1 | 0.007881652 | not include |  |
| TAA | RPA2 | 0.006711468 | not include |  |
| TAA | TUBB | 0.00631032 | not include |  |
| TAA | RPA3 | 0.005992564 | not include |  |
| TAA | BAG3 | 0.005913225 | not include |  |
| TAA | ARRB2 | 0.005820546 | not include |  |
| TAA | TRIM28 | 0.005550868 | not include |  |
| TAA | ABL1 | 0.005416963 | not include |  |
| TAA | CALM1 | 0.00513294 | not include |  |
| TAA | HSPB1 | 0.005131989 | not include |  |
| TAA | PRKCA | 0.004989527 | not include |  |
| TAA | COPS6 | 0.00462734 | not include |  |
| TAA | RUVBL1 | 0.004499802 | not include |  |
| ASH | TXN | 0.231514028 | include |  |
| ASH | HP | 0.073825469 | include |  |
| ASH | MMP9 | 0.056686679 | include |  |
| ASH | YWHAQ | 0.052538736 | not include |  |
| ASH | GRB2 | 0.044341615 | not include |  |
| ASH | MYC | 0.038102834 | not include |  |
| ASH | CUL1 | 0.034393827 | not include |  |
| ASH | CSNK2A2 | 0.026813689 | not include |  |
| ASH | HDAC1 | 0.025802891 | not include |  |
| ASH | HSP90AB1 | 0.02044351 | not include |  |
| ASH | CD40LG | 0.018519545 | include |  |
| ASH | CSNK1E | 0.018217694 | not include |  |
| ASH | TRAF2 | 0.017971302 | not include |  |
| ASH | PRKCA | 0.017614237 | not include |  |
| ASH | MAPK3 | 0.016968353 | not include |  |
| ASH | HSPA8 | 0.016388822 | not include |  |
| ASH | PAN2 | 0.015699159 | not include |  |
| ASH | BAG3 | 0.015287084 | not include |  |
| ASH | TCF4 | 0.0151421 | not include |  |
| ASH | EEF1A1 | 0.014282295 | not include |  |
| ASH | HNRNPD | 0.014260316 | not include |  |
| ASH | NCL | 0.012919532 | not include |  |
| ASH | ILF3 | 0.012312175 | not include |  |
| ASH | ARRB2 | 0.012247216 | not include |  |
| ASH | HSPA9 | 0.012144107 | not include |  |
| ASH | TUBB | 0.012044522 | not include |  |
| ASH | RPA1 | 0.012038275 | not include |  |
| ASH | RIOK2 | 0.011552218 | not include |  |
| ASH | FYN | 0.011180575 | not include |  |
| AA | MYH11 | 0.038456322 | include |  |
| AA | SMURF1 | 0.008789996 | not include |  |
| AA | EFEMP2 | 0.008402572 | include |  |
| AA | VCAM1 | 0.006977614 | not include |  |
| AA | RPA2 | 0.005328834 | not include |  |
| AD | GABARAPL2 | 0.053023683 | not include |  |
| AD | YWHAE | 0.0464928 | not include |  |
| AD | CDK4 | 0.041041964 | not include |  |
| AD | CDK1 | 0.03457681 | not include |  |
| AD | TARDBP | 0.030660038 | not include |  |
| AD | PCNA | 0.024676458 | not include |  |
| AD | SIRT1 | 0.023020062 | not include |  |
| AD | PPP2CB | 0.018402351 | not include |  |

**Table S4. Newly-published aneurysm-gene association of candidate drivers**

| Aneurysm subtype | Candidate driver | Source of supporting article | Species |
| --- | --- | --- | --- |
| CA | JUN | PMID: 30831289  PMID: 29328431 | Human |
| AAA | CSNK2A2 | PMID: 29439675 | Human |
| TAA | MYC | PMID: 23825360 | Human |
| TAA | TP53 | PMID: 25266234 | Human |
| ASH | MAPK3 | PMID: 34185228 | Human |
| ASH | MYC | PMID: 29884860 | Human |
| AA | VCAM1 | PMID: 32777344 | Mouse |
| AD | CDK1 | PMID: 31721906 | Human |
| AD | SIRT1 | PMID: 26376991 | Human |
